# Cannabinoids in Combination with Conventional Breast Cancer Therapies: Mechanistic Insights and the Gap to Clinical Translation

**DOI:** 10.3390/cancers18050761

**Published:** 2026-02-27

**Authors:** Anja Bizjak, Uroš Potočnik, Helena Čelešnik

**Affiliations:** 1 Center for Human Genetics & Pharmacogenomics, Faculty of Medicine, University of Maribor, Taborska ulica 8, 2000 Maribor, Slovenia; anja.bizjak2@um.si (A.B.); uros.potocnik@um.si (U.P.); 2 Faculty of Chemistry and Chemical Engineering, University of Maribor, Smetanova ulica 17, 2000 Maribor, Slovenia; 3 Department for Science and Research, University Medical Centre Maribor, Ljubljanska ulica 5, 2000 Maribor, Slovenia

**Keywords:** cannabinoids, CBS, breast cancer, chemotherapy, hormonal therapy, immunotherapy

## Abstract

Current treatments for breast cancer (BC) include surgery, radiation, chemotherapy, targeted therapy, hormonal therapy, and immunotherapy. However, adverse effects such as pain, nausea, cardiotoxicity, and neuropathy have prompted interest in complementary approaches. Cannabinoids (CBS), particularly cannabidiol and delta-9-tetrahydrocannabinol, are already used by cancer patients for symptom relief, and preclinical studies in cell culture and mouse models suggest additional therapeutic potential at the cellular level: combining CBS with chemotherapy may sensitize tumour cells to chemotherapeutic agents, inhibit tumour proliferation, and increase apoptosis. In murine models, such combinations may also mitigate chemotherapy-induced cardiotoxicity by enhancing antioxidant activity, modulating cannabinoid receptor signalling to reduce pro-inflammatory markers, and restoring mitochondrial function in myocytes. In addition, CBS may augment hormonal therapy in estrogen receptor-positive (ER+) BC cells, primarily via aromatase inhibition and modulation of ER and EGR3 signalling. Notably, evidence on combining CBS with targeted therapies in BC is lacking, while studies of CBS–immunotherapy combinations have been conducted in non-BC cancers; in BC, they are scarce and limited to in vitro models. This represents a key area for future research, particularly given the heterogeneity across non-BC cancers, where CBS–immunotherapy combinations have demonstrated mixed effects, both beneficial and detrimental (e.g., reduced response rates and overall survival), with the underlying mechanisms remaining unclear. Translation of these findings into clinical practice faces several challenges. Although over 120 CBS have been identified, only a few are well-characterized. CBS exhibit diverse mechanisms and effects, including potential adverse outcomes and interactions with conventional therapies (e.g., effects on chemotherapeutic drug metabolism). Variability among BC cells may also result in differing responses to the same therapeutic combinations. Future research should delineate the effects of individual CBS in combination strategies and prioritize well-controlled, standardized clinical studies to build on in vitro and animal data, while also exploring genetically informed personalized approaches. Ultimately, clinical guidelines specifying CBS type, formulation, and delivery are needed.

## Simple Summary

Breast cancer treatment relies on chemotherapy, hormonal therapy, targeted therapy, and immunotherapy, all of which may be associated with treatment-limiting adverse effects and heterogeneous responses. Cannabinoids are substantially used by breast cancer patients for symptom control, and preclinical studies suggest that cannabinoids may interact with established anti-cancer therapies through multiple biological mechanisms, potentially influencing treatment efficacy and toxicity. The purpose of this review is to critically summarize current preclinical evidence on the combination of cannabinoids with conventional breast cancer treatments, with emphasis on mechanistic insights. The evidence primarily focuses on three types of interactions: sensitization (enhancing conventional treatment) in vitro, antagonism (interference with therapy) in preclinical and clinical studies, and toxicity modulation, including side effects in patients. By identifying current limitations and gaps in knowledge, this review emphasizes the need for carefully designed translational and clinical studies evaluating cannabinoid use in breast cancer therapy.

## 1. Introduction

Breast cancer (BC) is one of the most common cancers worldwide and, in 2022, was the leading cancer in women across 157 of 185 countries, accounting for 670,000 deaths. Treatment for BC is typically a combination of surgery, radiation, and medication [1,2]. While these therapeutic approaches are widely used for BC, their adverse effects often lead to severe symptoms including pain, nausea, fatigue, loss of appetite, and sleep disturbances, which can significantly affect quality of life. In many cases, these symptoms are multiple and can have devastating results [3,4].

Among cancer patients, including BC patients, there is a substantial use of cannabinoids (CBS) [5,6]. Observational studies and structured surveys reported that many individuals initially experimented with CBS recreationally but began regular medicinal use after undergoing chemotherapy or surgery, or when their cancer progressed, to manage the aforementioned adverse symptoms [5,7,8]. Consequently, understanding CBS active compounds and their interactions with cancer therapies is increasingly important.

CBS exhibit diverse pharmacological effects, including receptor-dependent and receptor-independent mechanisms [9,10,11,12]. Receptor-dependent CBS mechanisms include cannabinoid receptor 2 (CB2)-mediated modulation of cancer cell proliferation and apoptosis and cannabinoid receptor 1 (CB1)-mediated inhibition of cancer cell invasion and metastasis in preclinical models [11,12]. Among receptor-independent mechanisms, studies have reported anti-inflammatory, analgesic, antiproliferative, and pro-apoptotic properties [9,10]. Among more than 100 CBS identified in the cannabis plant, delta-9-tetrahydrocannabinol (THC) and cannabidiol (CBD) are the most extensively studied. THC is the main psychoactive compound, whereas CBD is non-psychoactive and may counteract some of THC’s effects [13,14]. Additionally, research on neuronal mouse models has indicated CBD’s anxiolytic and antipsychotic properties, further contributing to its therapeutic potential [15].

Given the rising use of CBS, a growing number of studies are now examining their potential interactions with standard BC treatments.

## 2. CBS Combined with Chemotherapy: Symptom Relief, Cardiac Protection, and Tumour Suppression

### 2.1. Pain Management

Emerging studies suggest that CBS may help alleviate some of the treatment-related symptoms in BC, particularly in pain management, nausea reduction, and overall quality of life improvement [16,17]. Studies in murine neuronal models have shown that CBD, or the combination of CBD and THC, can alleviate peripheral neuropathy associated with paclitaxel, a commonly used chemotherapeutic agent for BC [18,19,20]. Mechanistically, in vitro studies on BC cells identified activation of the 5-HT1A receptor system as a pathway through which CBD alleviates PTX-induced neuropathic pain [19,20]. The 5-HT1A receptor system is part of the serotonergic pathway and functions as a G-protein-coupled receptor involved in the regulation of mood, pain perception, and neuroprotection [21].

### 2.2. Alleviation of Cardiotoxicity

It is well established that a considerable number of antitumour pharmaceuticals, including those commonly employed in BC treatment, such as doxorubicin, cisplatin, and epirubicin, exhibit toxic effects on cardiac muscle [22]. Cardiotoxicity has the potential to lead to irreversible adverse effects in patients, particularly in the context of chemotherapy and targeted therapies [22]. Chemotherapeutic drugs are frequently associated with oxidative stress-induced cardiotoxicity, which can ultimately result in congestive heart failure. Additionally, targeted biological therapies, such as HER2 inhibitors like trastuzumab or pertuzumab, have been reported to contribute to left ventricular dysfunction and other cardiac complications [22,23,24]. Consequently, researchers have been actively investigating cardioprotective agents, although in most of the cited studies, cardioprotective effects and tumour-related outcomes were not evaluated simultaneously within the same experimental model. Recent studies have suggested that CBS may offer protective effects against treatment-induced cardiomyopathy [25,26,27]. Furthermore, synergistic effects have been suggested when CBS are combined with chemotherapy [28,29,30], although only one study supported that with formal synergy testing [30]. However, several studies have reported treatment enhancement when chemotherapy was combined with CBD [27,31,32,33].

Among chemotherapeutic agents that have been demonstrated to induce cardiomyopathy, the anthracycline doxorubicin is notable for causing increased nitric oxide (NO) production [27,34]. NO is generated by isoforms of NO synthase, and one isoform, inducible NO synthase (iNOS), has been found in greater quantities in cells treated with doxorubicin.

**Figure 1:**
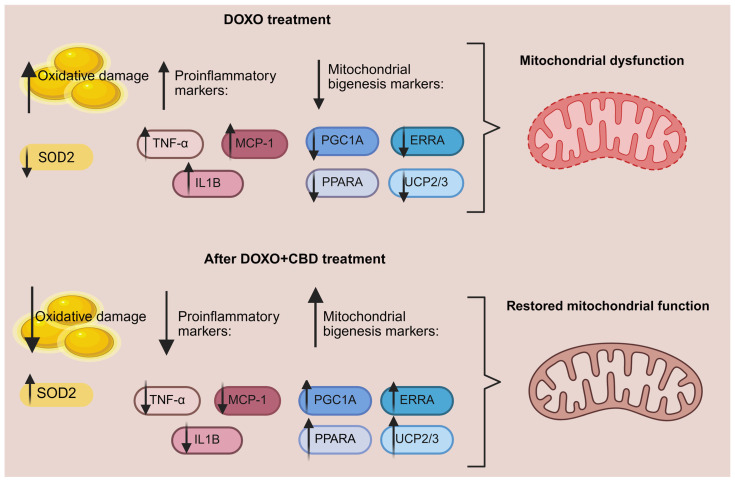
Proposed mechanism of restored myocyte mitochondrial function after doxorubicin (DOXO) + CBD treatment [27,35] (Created in BioRender. Bizjak, A. (2026). https://BioRender.com/qtm2746).

Importantly, studies combining doxorubicin treatment with CBS have indicated therapeutic potential (Figure 1).

In murine studies by Tabatabaei et al., administration of CBD in conjunction with doxorubicin was associated with a substantial reduction in tumour size [27]. The combination of CBD and doxorubicin also increased the expression of superoxide dismutase (SOD2), an enzyme with antioxidant activity [27]. Another murine study reported decreased iNOS expression when doxorubicin was combined with CBD [35]. The combination also showed alterations in markers related to mitochondrial biogenesis, which could be relevant, since doxorubicin-induced cardiotoxicity is associated with mitochondrial dysfunction. Specifically, doxorubicin treatment in mice resulted in decreased myocardial mitochondrial copy number and showed a secondary pro-inflammatory response through increased expression of *TNFA*, *IL1B* and *MCP-1.* Doxorubicin treatment also resulted in downregulation of mitochondrial biogenesis markers, such as *PGC1A*, *PPARA*, *ERRA* and *UCP2*, *UCP3*. These genes are central regulators of mitochondrial function and are also implicated in controlling reactive oxygen species (ROS) production and inflammatory responses [36,37,38,39]. Notably, CBD largely prevented these changes in mice, suggesting that it plays a role in restoring mitochondrial function and energy metabolism in cardiac cells [35]. Furthermore, murine studies on CB1 antagonists have indicated that CB1 receptors can exert an effect on the mitochondria. Blocking CB1 receptors mitigates myocardial infarction damage by inhibiting NF-kB-mediated pro-inflammatory signalling, decreasing cytokines such as TNF-α, IL-1β, and IL-6, and restoring antioxidant defences, including SOD, glutathione (GSH), and catalase levels [40].

Overall, cardiotoxicity represents a serious and clinically relevant adverse effect of several cancer treatments and can significantly impact patient morbidity and long-term survival. While preclinical in vivo studies have reported cardioprotective effects of CBS in murine models, corresponding clinical studies are currently lacking, making the clinical relevance of these findings uncertain.

### 2.3. Apoptosis and Tumour Size

An in vitro study by Patel et al. using triple-negative MDA-MB-231 BC cells investigated the combined effect of CBD and doxorubicin. The study reported a significant decrease in the protein expression of NF-Κb [32], a transcription factor that plays an important role in inducing BC cell proliferation and invasiveness [41]. Another important regulator of proliferation and BC invasiveness is mTOR [42]. Patel’s study demonstrated that combining doxorubicin with CBD significantly downregulated mTOR expression in MDA-MB-231 xenograft tumours, highlighting the anti-proliferative effect of this treatment [32]. Moreover, compared to doxorubicin alone, the CBD combination therapy increased the levels of pro-apoptotic markers BAX and Caspase 9. The combination of CBD with doxorubicin also led to a significant reduction in tumour volume in xenograft models [32]. In these experiments, combination effects were evaluated using selected concentrations of CBD-loaded extracellular vesicles together with fixed doses of doxorubicin. While enhanced antitumor effects were observed compared to doxorubicin alone, no formal synergy models were applied, and therefore synergistic interactions cannot be concluded.

**Figure 2:**
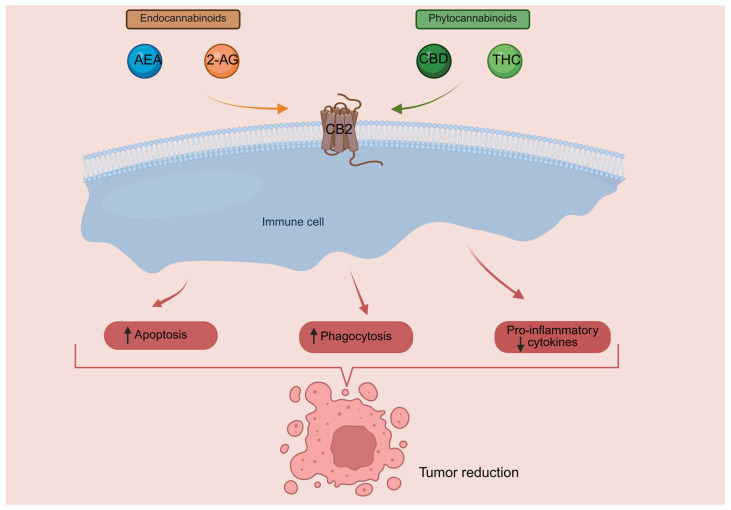
Proposed schematic of CB2 receptor-mediated immunomodulatory effects of endocannabinoids and phytocannabinoids in BC [46]. (Created in BioRender. Bizjak, A. (2026). https://BioRender.com/qtm2746) AEA: Anandamide; 2-AG: 2-Arachidonoylglycerol; CBD: Cannabidiol; THC: Δ^9^-Tetrahydrocannabinol.

Another study investigated the role of CB2 in BC [11]. CB2 receptors are highly expressed on immune cells, and their activation modulates immune cell behaviour, including inflammatory signalling and macrophage-mediated tumour clearance [43,44]. CB2-selective agonist JWH-133 has been demonstrated to exert antitumoral effects in a mouse model of breast cancer [45]. A schematic overview of CB2-mediated signalling is shown in Figure 2.

**Figure 3:**
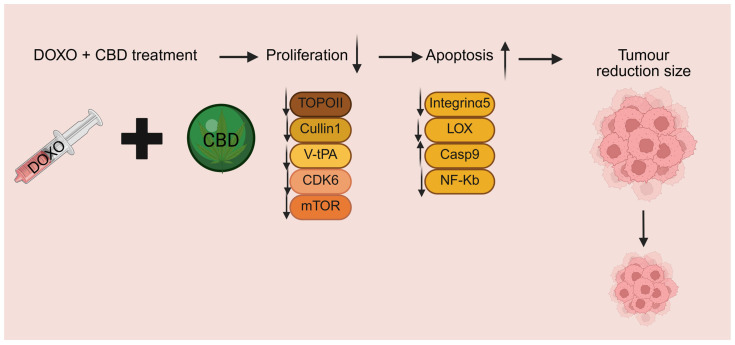
Enhancement of tumour suppression and promotion of apoptosis by CBD and chemotherapy [29,30,32,33]. (Created in BioRender. Bizjak, A. (2026). https://BioRender.com/qtm2746) TOPO: topoisomerase II; V-tPA: V-type proton ATPase; CDK6: Cyclin Dependent Kinase 6; LOX: Lysyl oxidase; Casp9: Caspase 9; NF-Κb: nuclear factor kappa B; mTOR: mechanistic target of rapamycin kinase.

In the study by Song Q et al., high tumour CB2 expression was associated with better survival of BC patients, and BC cells overexpressing CB2 showed increased sensitivity to cisplatin, doxorubicin and docetaxel [11]. CB2 overexpression was associated with reduced Ki67 expression and decreased proliferation, along with increased apoptosis, possibly mediated by suppression of the PI3K/Akt/mTOR signalling pathway. In line with these findings, a substantial reduction in tumour size was observed in murine models, when doxorubicin was combined with CBD, and the results were better compared to the doxorubicin-alone group [27]. Consistently, a higher induction of apoptosis in CBD + doxorubicin treated groups compared to doxorubicin alone was observed [27]. A study assessing the sensitization effect of CBD to doxorubicin treatment found that CBD pre-treatment reduced integrin-alpha 5 and Lysyl oxidase expression while increasing caspase-9 levels in BC cells, suggesting a potential pathway for enhancing apoptotic response and treatment efficacy (Figure 3) [33].

In the context of different BC subtypes, another study investigated the combination of CBD with doxorubicin and paclitaxel in estrogen receptor (ER)-positive BC cells and triple-negative BC (TNBC) cells [29]. CBD in solution (CBDsol) enhanced the activity of Paclitaxel in both BC cell lines, demonstrating a positive effect on tumour suppression, whereas paclitaxel alone was less effective. A moderate additive effect of paclitaxel and CBD was suggested that could be associated with increased apoptosis in cancer cells [29]. Similar interactions were observed when CBDsol was combined with doxorubicin, with the effects being far more pronounced in the combination treatment than with either CBDsol or doxorubicin alone. These findings support the hypothesis that CBS may enhance chemotherapy-induced cell death [29].

It should be noted that results obtained in simplified in vitro models do not fully reflect the biological complexity of human tumours, including the influence of serum components, protein binding, and tumour microenvironment.

A bioinformatic study identified three key hub genes linked to TNBC [29]. Identified hub genes—*RPL7A*, *NHP2L1*, and *PSMD11*—are involved in ribosomal formation, protein synthesis, splicing factors, and proteasome function, suggesting potential relevance to TNBC development and progression [47]. Promising interactions of CBD analogues with these candidates were identified, suggesting a potential therapeutic avenue for TNBC treatment, especially in combination with chemotherapy drugs like doxorubicin. However, these in silico findings are hypothesis-generating and require experimental validation. Additionally, a proteomics study reported synergistic interactions between CBD and five chemotherapeutic drugs (doxorubicin, docetaxel, paclitaxel, irinotecan, and vinorelbine) in ER-positive MCF-7 BC cells, highlighting potential opportunities to enhance BC treatment [30]. The synergistic effect of CBD with chemotherapy increased apoptotic cell numbers in MCF-7 cells, reinforcing its potential as an adjunct therapy. The study also suggested a new mechanism for the cytotoxic effects of CBD on cancer cells, involving inhibition of topoisomerase IIβ and IIα, cullin 1, V-type proton ATPase, and cyclin dependent kinase 6, which are essential for cancer cell survival [30,48,49,50,51]. This led to sabotaged energy production and impaired mitochondrial translation, further weakening cancer cells (Figure 3) [30].

### 2.4. Cautions Regarding Combining CBD with Chemotherapy

However, caution is necessary, as in vitro studies indicate that under certain circumstances, CBS may interfere with chemotherapy effectiveness. A recent study using triple-negative MDA-MB-231 cells reported that the effect of CBD on cisplatin depends on serum concentration [52]; under high-serum conditions (10% FBS), CBD significantly reduced cisplatin toxicity, suggesting that serum components may influence CBD’s interaction with chemotherapy drugs. This observation may be partly explained by findings indicating that both CBD and THC exhibit high plasma protein binding. Reports on serum dependence and protein binding are highly relevant as it can fundamentally change how CBS behave in vitro versus in vivo, therefore limiting interpretations of in vitro findings. Additionally, in another study using TNBC cells, a higher CBD dosage (2.5 µM) in combination with doxorubicin produced an antagonistic effect [33]. These findings contrast with the synergistic responses reported in the proteomics-based study by Alsherbiny et al., which evaluated the synergy of CBD in combination with multiple chemotherapeutic agents in ER-positive BC cells [30]. These discrepancies highlight the combination’s reliance on particular experimental conditions and further underscore the complexity of CBD–chemotherapy interactions. The reported dose-dependent and context-specific effects, together with the absence of formal synergy quantification, suggest that these interactions may be hard to predict. Together, these findings highlight the need for caution when CBD is added as a complementary therapy [33,52].

## 3. CBS Combined with Hormonal Therapy

More than half of BC tumours express ERα and are responsive to hormonal therapy [53]. The main treatment strategy consists of aromatase inhibitors, which deprive ER-positive BC cells of estrogen-driven growth signals by inhibiting the aromatase enzyme [54]. The aromatase inhibitors include Exemestane, Letrozole, and Anastrozole. Steroidal aromatase inhibitors or type I inhibitors (e.g., Exemestane) bind covalently and irreversibly to the aromatase enzyme [55,56]. In contrast, non-steroidal aromatase inhibitors or type II inhibitors, such as Anastrozole and Letrozole, bind non-covalently and reversibly [56,57,58,59,60]. In addition, selective ER modulators, such as Tamoxifen and selective ER degraders, such as Fulvestrant, are used in ER-positive BC. CBS may play a role as a single agent or in combination with hormonal therapy by influencing estrogen signalling and related pathways instead of or in addition to androgen receptors [61].

### 3.1. The Effects of CBS as a Single Agent on ER-Positive BC Cells

In vitro studies using ER-positive cells suggest that CBD acts as an ERα antagonist with inverse agonist properties, while THC disrupts estrogen signalling by upregulating ERβ [61,62]. While these effects were observed under defined in vitro conditions and may thus be context-dependent and cell-line restricted, they are of interest because ERβ is thought to counteract the proliferative effects of ERα in BC cells, potentially providing a therapeutic advantage.

The study by Amaral et al. investigated the effects of different CBS (anandamide, CBD, and THC) on ER-positive BC cells [63]. The results showed that these CBS disrupt the cell cycle and induce cell death in ER-positive BC cells through distinct mechanisms. Anandamide and THC primarily induce apoptotic cell death, whereas CBD promotes both autophagy and apoptosis, leading to enhanced cancer cell death. Notably, all three CBS inhibit aromatase activity and reduce ERα expression, both of which are crucial for estrogen-driven cancer growth. Among the compounds tested, CBD demonstrated the most significant impact, as it not only exhibited high anti-aromatase activity but also weakened ERα signalling by decreasing CYP19A1 mRNA and protein levels, thereby impairing aromatase synthesis [63]. The CYP19A1 gene encodes aromatase, which catalyzes the final step of estrogen biosynthesis; its elevated expression is associated with poor prognosis and has been proposed as a potential diagnostic and prognostic marker in BC [64,65]. This dual action on aromatase and estrogen signalling highlights CBD’s potential as a multitarget therapeutic agent in ER-positive BC. The study hypothesized that different CBS may reach therapeutic targets more efficiently than the drug tamoxifen due to their bioavailability, lower risk of toxicity and stronger binding affinities [63].

A study by Daoui et al. applied computational strategy and virtual screening of multiple CBS and identified CBD, anandamide, and THC for strong effects on ER and aromatase inhibition [66]. Also, it reported that CBS may act as reversible inhibitors of the Epidermal Growth Factor Receptor Tyrosine Kinase Domain, a crucial regulator of cell division and survival [66,67]. While the authors did not perform experimental combination treatments, they did use Molecular Dynamics simulations to investigate the interactions.

### 3.2. The Effects of CBS in Hormonal Therapy Combination

Several studies have investigated the effects of CBS in combination with hormonal therapy (Table 1).

Buijs et al. investigated the influence of CBD oil use in patients receiving Tamoxifen therapy [68]. This question is clinically relevant because discontinuation rates for Tamoxifen due to adverse effects have been reported to be as high as 40% [68,69]. Tamoxifen is a prodrug that is converted into its active metabolite, endoxifen, which was measured in the study in [70]. Use of CBD oil was associated with a reduction in side effects such as hot flashes and arthralgia, as well as improved health-related quality of life scores [68]. Although endoxifen levels decreased slightly, they remained within bioequivalence limits, indicating that CBD oil does not meaningfully interfere with Tamoxifen’s therapeutic effect, only with its side effects [68]. Still, this effect was observed in a small sample size, and additional studies with larger cohorts and long-term follow-up are needed to better support the findings. Nonetheless, an in vitro study reported similar findings in ER-positive BC cell lines treated with Tamoxifen and CBD [71]. The Tamoxifen–CBD combination further reduced cell viability in the T-47D cell line. The effect of combination was not similar in another ER-positive BC cell line, MCF-7, which exhibits lower expression of progesterone receptor (PR). It was therefore hypothesized that the differences in PR expression level may influence cellular responses to combination treatment [71,72].

Another study explored the combination of CBD with aromatase inhibitors on MCF-7aro cells, which overexpresses aromatase, and found that while CBD did not notably enhance the effects on non-steroidal aromatase inhibitors, it significantly potentiated the pro-apoptotic effects of the steroidal aromatase inhibitor Exemestane [73]. The combination of CBS and Exemestane impaired ER activation and abolished the estrogen-like effect of Exemestane. Specifically, the combination of Exemestane and CBD led to a significant reduction in ERα-regulated signalling, supporting an anti-proliferative mechanism. Furthermore, treatment of ER-positive MCF-7aro cells with Exemestane and CBD resulted in decreased androgen receptor (AR) expression. While Exemestane and CBD individually activated caspase-7, their combination further enhanced the activation of apoptosis compared with either compound alone. In contrast, no notable effect was observed for combination of CBD with Anastrozole or Letrozole [73]. Moreover, CBD in combination with Exemestane also inhibited activation of the extracellular signal-regulated kinase 1/2 (ERK1/2) pathway, promoting apoptosis [73]. The ERK1/2 pathway is a key effector of the MAPK cascade, which is abnormally activated in a number of human cancers [74,75,76]. The study also analyzed mRNA transcript levels of the EGR3 gene, which can be induced following ERK1/2 pathway activation [61,77,78]. Proteins of the EGR family are vital for a variety of biological processes, including muscle and lymphocyte development, endothelial cell growth, migration, and neuronal development [79]. The EGR3 family member is involved in estrogen receptor signalling and can act as a mediator of ERα-driven transcriptional programmes that promote tumour cell survival [79]. EGR3 may play a role in hormonal therapy responsiveness, as upregulation of EGR3 has been reported in tamoxifen-resistant ERα-positive breast cancer, with higher expression in MCF-7R and T47D-R cells compared with their parental ER-positive cell lines. Interestingly, in a study using MCF-7aro cells treated with Exemestane, Exemestane alone did not decrease EGR3 transcription. However, a substantial decrease was observed when Exemestane was combined with CBD [73]. It has been postulated that CBD may circumvent weak estrogen-like effects of Exemestane by preventing EGR3 transcription, thereby further reducing estrogen signalling and reinforcing its pro-apoptotic effect [73,80]. Additionally, AR expression has been associated with increased proliferation and enhanced cancer cell survival in MCF-7-derived tamoxifen-resistant cells, underscoring the potential therapeutic relevance of AR downregulation [81]. Accordingly, the combination of Exemestane with CBD in BC cells has been suggested to play a role in overcoming endocrine resistance through the reduction in AR expression.

Another important observation concerns the selectivity of aromatase inhibitor–CBD combinations for cancer cells. Whereas the treatment with CBD and Exemestane reduced the viability of MCF-7aro cells, it did not affect the viability of non-tumorigenic, HFF-1 cells. This finding underscores the possible specificity and the potential therapeutic safety and relevance of the combination [73]. Nonetheless, selectivity toward cancer cells was inferred from a single non-tumorigenic line. The scope of normal cell testing is thus limited, necessitating further validation in a wider range of normal cells to confirm these observations.

A recent study investigated the estrogen receptor-mediated transcriptional activity of minor CBS [61]. Using VM7Luc4E2 reporter cells expressing either human ERα or ERβ isoforms, cannabinol was identified as the most promising minor cannabinoid, due to its multi-target activity across several pharmacologically relevant pathways in ER-positive BC. Cannabinol was also evaluated in the MCF-7aro cell line for its potential to enhance the anti-cancer efficacy of three clinically used aromatase inhibitors: Anastrozole, Letrozole and Exemestane [82,83]. The greatest enhancement was observed in a combination of cannabinol with Exemestane [83]. The combination reduced ERα protein levels at both tested concentrations, whereas a decrease in ERα transcription was observed only at the lower dose, suggesting a dose-dependent effect and post-transcriptional regulation. At both concentrations, the combination also significantly decreased CYP19A1 transcription, aromatase expression, and AR gene transcription compared with Exemestane alone. Nevertheless, in contrast to earlier findings with the CBD-Exemestane combination, the cannabinol-Exemestane combination did not significantly enhance apoptosis [73,83]. Instead, it showed only a trend toward increased caspase-7 activation compared to Exemestane alone, which did not reach statistical significance in the reported data [83].

In a pilot study evaluating CBD in BC patients receiving hormonal therapy, a clinically meaningful reduction in worst pain was observed in a subset of participants [84]. CBD treatment was associated with reductions in several unstimulated pro-inflammatory cytokines, including IL-1β, TNF-α, IL-17A, IL-18, and IFN-γ, consistent with a potential anti-inflammatory mechanism [85]. Notably, CBD was administered alongside Anastrozole, Exemestane, or Letrozole. However, outcomes were not stratified by individual aromatase inhibitor, limiting conclusions regarding drug-specific interactions. Baseline anxiety was identified as a potential modifier of CBD response, suggesting that interindividual patient characteristics may contribute to the observed heterogeneity in clinical outcomes.

**Table 1:** The effects of CBS on hormone therapy [68,71,73,83].

Ref.	Model	Combination	Key Outcomes of Combination Treatment
[68]	BC patients	CBD + Tam	- reduced side effects - improved health-life quality - CBD did not interfere with Tam’s efficiency
[71]	T-47D (BC ER+ cell line) MCF-7 (BC ER+ cell line)	CBD + Tam	- reduced T-47D cell viability, but not MCF-7 (PR may modulate response)
[63,73]	MCF-7aro (BC ER + cell line) HFF-1 (non-tumorigenic cell line)	CBD + Ana CBD + Exe CBD + Let	- CBD + EXE most promising - CBD + EXE induction of apoptosis: activation of caspase-7 reduced viability - CBD + EXE anti-proliferative effect: impaired ERα activity downregulation of ERα-regulated gene *EGR3* - CBD + EXE overcome resistance pathways: Downregulation of AR signalling - CBD + EXE selectivity: reduction in MCF-7aro but not HFF-1
[73,83]	MCF-7aro (BC ER+ cell line)	CBN + Ana CBN + Exe CBN + Let	- CBN + EXE most promising - CBN + EXE slight induction of apoptosis: Trend toward increase in caspase-7 activation, but not statistically significant - CBN + EXE anti-proliferative effect reduced ERα protein levels decline in Erα transcript levels at lower concentrations, possible post-transcriptional regulation downregulation of Erα-regulated gene *EGR3* decreased CYP19A1 transcription and aromatase expression - CBN + EXE overcome resistance pathways: suppression of AR signalling reduction in worst pain reductions in pro-inflammatory cytokines
[84]	BC patients	CBD + Ana CBD + Exe CBD + Let	- reduction in worst pain - reductions in pro-inflammatory cytokines

Ana: anastrozole; CBD: cannabidiol; CBN: cannabinol; ER: estrogen receptor; EXE: Examestane; Let: Letrozole; MCF-7: ER-positive human BC cell line. MCF-7aro: ER-positive human BC cells stably transfected with aromatase gene; T-47D: ER-positive human BC cell line; HFF-1: Human Foreskin Fibroblast-1 non-tumourigenic cell line.

## 4. CBS Combined with Targeted Immunotherapy in BC Is an Understudied Area

### 4.1. CBS and Targeted Therapy

Targeted therapies, including tyrosine kinase inhibitors (TKIs) such as Tucatinib, Neratinib, and Lapatinib, as well as Trastuzumab, are central to the management of HER2-positive breast cancer. Despite their clinical importance, no studies have explored the combination of CBS with these targeted therapies, highlighting a significant gap and an area in need of future research. This is particularly relevant for Trastuzumab, which is associated with cardiotoxicity [22,23,24], given that limited in vitro and in vivo evidence suggests that CBS may modulate cardiac injury both in general cardiac stress models and in chemotherapy-associated settings [25,27,86]. CBS have been reported to reduce the cardiotoxicity caused by the chemotherapeutic doxorubicin by acting on pathways related to oxidative stress and cellular stress responses [25,27]. As some of the injury mechanisms involved in trastuzumab-induced cardiotoxicity are similar, there is a possibility that the protective pathways may overlap [22,23,24]; however, no studies have directly investigated this interaction. Investigating whether CBS could modulate effects on heart damage and influence targeted therapeutic outcomes would be of considerable interest. One study reported high expression of CB2 in HER2+ BC and its association with poor prognosis [87,88]. HER2-targeted therapies, partially depend on immune-mediated mechanisms [89]. In BC models, CBS signalling has been linked to modulation of tumour microenvironment cytokine networks and HER2-related immune pathways, suggesting that CBS-mediated immune regulation could theoretically influence therapeutic responses in specific biological contexts [87,89,90]. Given that CBS have been reported to modulate immune cell function via CB1 and CB2 receptors [11,12], as well as exert receptor-independent effects on tumour cells [10], potential interactions between CBS and HER2-targeted therapies cannot be excluded.

### 4.2. The Uncertain Role of CBS in Immunotherapy

Immunotherapy has emerged as a powerful approach in cancer treatment [91,92,93]. Nevertheless, despite its successes, resistance and immune-related adverse effects remain challenging [94,95,96].

Emerging evidence suggests that CBS signalling may influence tumour–immune interactions by modulating cytokine production, immune cell activation, and the tumour microenvironment, processes that are central to immune checkpoint blockade [9,87,88,90]. A recent systematic review evaluating CBS use during immune checkpoint inhibitor therapy across cancers highlighted potential immunomodulatory effects of CBS and the need to better understand their impact on therapeutic responses [97]. The authors stress that prospective studies with standardized CBS reporting are needed to clarify potential interactions with immune checkpoint inhibitor therapy.

Several studies have explored the effects of CBS use in patients with different cancers undergoing immunotherapy [98,99,100]. However, the findings remain inconclusive, and reports specifically for BC are scarce. Findings across cancers are mixed, with some studies suggesting that CBS do not compromise immunotherapy efficacy, whereas others highlight concerns regarding decreased response rates and overall survival [98,99,100]. Interpretation of these findings is limited by substantial heterogeneity in clinical studies, including mixed cancer types, low and non-standardized cannabis exposure, and diverse formulations [98,101]. Additionally, mechanistic studies are largely based on in vitro models that do not capture the biological and immunological complexity of human tumours [99,100]. These seemingly paradoxical findings may further be explained by the immunomodulatory effects of CBS, which can alter the balance and functional state of immune effector cells and cytokine signalling pathways that are critical for the efficacy of immune checkpoint inhibitors [102,103,104,105].

#### 4.2.1. Combination of CBS and Immunotherapy for BC: Implications for Programmed Death-Ligand 1 Regulation

Atezolizumab is a monoclonal antibody that targets programmed death-ligand 1 (PD-L1). It was previously used for the treatment of TNBC but was withdrawn in some countries in 2021 after it was shown not to confer a survival benefit in the overall population [106,107]. However, a 2024 in vitro study by Kim et al. reported that CBD can enhance the efficacy of atezolizumab in TNBC by upregulating PD-L1 expression via the cGAS-STING pathway in four PD-L1-positive TNBC cell lines: MDA-MB-231, BT20, 4T1 and EMT6 [100]. It is important to note that the cGAS-STING pathway plays a significant role in the innate immune response by helping dendritic cells and other immune cells to detect tumour-derived DNA. Upregulation of the pathway boosts immune responses, potentially improving the therapeutic effects of PD-L1 inhibitors such as atezolizumab [100].

Another important immunotherapy used in TNBC treatment is Pembrolizumab, a humanized monoclonal immunoglobulin directed against the human cell surface programmed cell death-1 receptor (PD-1) [108,109]. We were unable to find any studies investigating the combination of Pembrolizumab with CBS in BC. However, two studies have evaluated the impact of CBS on Pembrolizumab treatment in other cancers [101,110]. Although these are not BC studies, findings from other solid tumours may offer valuable insights into potential drug interactions and their implications for BC patients.

One study included both preclinical and clinical data for evaluating the effect of THC on Pembrolizumab treatment in non-small cell lung cancer. In the CT26 tumour-bearing murine model, THC did not compromise the antitumor activity of Pembrolizumab therapy, and in patients, time to tumour progression was comparable between CBS users and non-users [101]. In contrast, a retrospective cohort study in patients with solid malignancies reported worse overall survival, progression-free survival, and disease control rates in those receiving Pembrolizumab with concomitant THC use [110]. These findings suggest that THC consumption may negatively influence treatment outcomes. Importantly, CBS exposure in this study included a broad and heterogeneous range of products and use patterns, prescribed and non-prescribed, with no standardization of formulation, dose, or frequency. This heterogeneity limits definitive conclusions and causal interpretation of CBS’s effect on imunotherapy.

A similar association with poorer outcomes was reported in another study that investigated CBS with Nivolumab, another PD-1 binding monoclonal antibody. Like Pembrolizumab, Nivolumab binds PD-1 at flexible loop regions that overlap with the PD-L1 binding site, although at a different location and to a different extent. In both cases, this effectively blocks PD-1/PD-L1 interaction [111]. Even though Nivolumab is not yet used in BC treatment, its role continues to evolve [112,113].

This study, which assessed the impact of CBS consumption through smoking or edibles in patients receiving Nivolumab treatment [98], found that the immunotherapy response rate was significantly lower in the CBS group, approximately 16%, compared to the non-CBS group, approximately 38%. CBS users also experienced shorter time to tumour progression and worse overall survival, which may be attributed to the immunomodulatory effects of CBS [98]. Similar to the aforementioned study, CBS exposure in this study was highly heterogeneous, limiting mechanistic interpretation. Because different CBS formulations and routes of administration yield distinct systemic concentrations and metabolite profiles, this heterogeneity limits definitive conclusion and causal interpretation of CBS’s effect on imunotherapy [114].

#### 4.2.2. Immunomodulatory Mechanism of CBS

The abovementioned clinical findings may be partly explained by the immunomodulatory effects of CBS. In multiple murine models, THC has been found to suppress antitumour T-cell immunity by downregulating JAK1-STAT signalling in T cells [102]. It has been observed that tumour-infiltrating T-cell expression of TNF-α and IFN-γ is reduced by THC, while the tumour expression of the anti-inflammatory cytokines IL-10 and TGF-β is upregulated [102,103]. Moreover, treatment of stimulated peripheral blood mononuclear cells from healthy donors with CBD has led to notable effects on immune and inflammatory regulation of Treg and Th17 cells. CBD has been reported to suppress extracellular expression of both anti- and pro-inflammatory cytokines in the PBMC inflammation model. Reduced secretion of pro-inflammatory cytokines has been observed under CBD treatment. The treatment also influenced regulatory T cells, showing dose-dependent modulation, with reductions observed at specific concentrations [104]. Furthermore, a study has shown that CBD can negatively modulate neutrophil migration, survival, chemotaxis and oxygen uptake [105]. Investigations in mast cells found that endogenous and synthetic CB1 agonists activate cannabinoid receptors, leading to upregulation of adenyl cyclase, chronically increasing cAMP, which suppresses their secretory responses [115,116].

## 5. Potential Combination of CBS with Experimental Photodynamic Therapy

**Figure 4:**
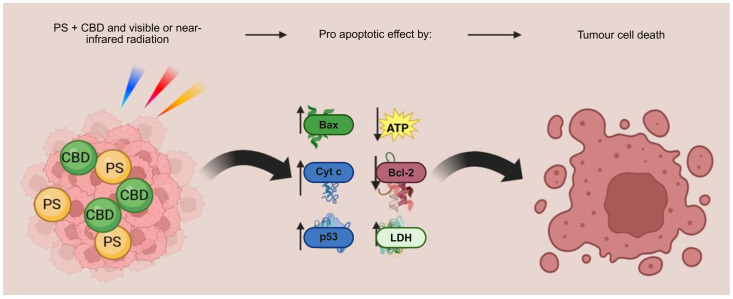
Photodynamic therapy (PDT) combination with CBD [123]. (Created by biorender.) PS: photosensitizer.

One of the newer, still exploratory, spatially targeted approaches is photodynamic therapy (PDT). PDT is a minimally invasive technique that uses visible or near-infrared radiation to activate a photosensitizer (PS), leading to the production of ROS and cell death [117]. The photodynamic effect relies on the simultaneous presence of a photosensitizer, oxygen, and specific light wavelengths, none of which are harmful on their own. However, when combined, they trigger cytotoxic effects selectively in the targeted tissue [117,118,119]. Based on the promising selectivity of the method and the challenges of treating BC, PDT has been explored both alone and in combination with CBS [120,121,122,123]. In multiple BC cell line models, MDA-MB-231 (TNBC), MCF-7 (ER+), and SK-BR-3 (HER2+), treatment with photodynamic particles resulted in a substantial decrease in cancer cell viability and significant increase in ROS production [124]. The study also reported G2/M cell cycle arrest in ER-positive MCF-7 cells, while TNBC cells (MDA-MB-231) accumulated primarily in the S phase, with only a modest increase in the G2/M phase. Another PDT study in MCF-7 and hTERT-HME1 cells showed elevated ROS production and upregulation of the apoptotic markers and caspases [121]. A study by Mokoena et al. investigated the combination of PDT and CBD in MCF-7 cells, showing some promising results (Figure 4) [123]. The combination resulted in morphological changes such as vacuolization, shrinking, and floating cells. In addition, the combination was also associated with apoptosis. These effects were supported by increased LDH release and reduced ATP levels, immunofluorescence evidence of upregulated pro-apoptotic proteins (BAX, TP53, Cyt c), and reduced anti-apoptotic BCL-2. However, it must be noted that PDT remains investigational in BC; its application is largely experimental and has not yet been established in routine clinical practice.

## 6. Perspectives and Future Directions

### 6.1. Potential Benefits

This review aimed to examine the mechanisms underlying the combination effects of CBS with current BC therapies. Several potential benefits of these combinations have been reported. While conventional therapies are often associated with high rates of adverse effects, in vivo and in vitro studies suggest these may be mitigated or mechanistically altered by CBS [7,20,68,73]. At the cellular level, combining CBS with chemotherapy or hormonal therapy has demonstrated promising outcomes, such as increased apoptosis, tumour reduction, and inhibition of proliferation [19,27,32,68,73,83]. Furthermore, while the aromatase inhibitor Exemestane has been reported to induce the upregulation of AR expression in MCF-7 cells, contributing to the development of resistance [83], CBD and cannabinol have been shown to downregulate AR signalling in BC cells, thereby negating the pro-survival effect that is typically associated with AR signalling [73,83]. Additionally, combining CBD with PTS (an experimental method of BC treatment) in vitro also demonstrated promising effects by a substantial increase in ROS generation in cancer cells and a significant reduction in BC cell viability [123].

### 6.2. Potential Risks of Harm

While combining CBS with current therapies may offer benefits, potential risks should also be considered, particularly regarding the availability and metabolism of conventional treatments. For instance, a prevalent limitation of chemotherapy in BC patients is anthracycline-induced cardiomyopathy [125,126]. Preclinical studies suggest that CBS combined with chemotherapy can be protective, increasing SOD2 expression, decreasing iNOS expression, and restoring mitochondrial function in mouse models [27,35]. However, other studies using recombinant human CYP450 enzymes and human liver microsomes have demonstrated that CBS can inhibit multiple CYP isoforms [127,128]. This is important, since CYP enzymes are responsible for the metabolism of a significant number of chemotherapy drugs [129,130]. All three major cannabinoids, THC, CBD and cannabinol, have been reported to inhibit CYP3A and CYP3A5 enzymes, with CBD showing the most potent inhibition [131]. Several chemotherapeutic agents commonly used in BC, including docetaxel, doxorubicin, and paclitaxel, are metabolized in part by CYP3A4/3A5 [129,130,132,133,134]. Thus, the inhibition of these enzymes may alter drug clearance and toxicity profiles. The clinical relevance of altered CYP activity is further underscored by pharmacogenetic studies showing that variability in hepatic CYP3A4 is associated with differences in doxorubicin clearance in BC patients [129]. Lower hepatic CYP3A4 expression was associated with reduced doxorubicin clearance. In addition, CBD has been associated with dose-dependent liver enzyme elevations in a small proportion of patients, which, particularly in combination with systemically metabolized anti-cancer therapies, may contribute to interindividual variability in treatment tolerance [135].

Moreover, clinical studies combining CBS-based substances with cancer treatment have reported variable outcomes. In BC patients receiving hormonal therapy, CBD does not appear to interfere with treatment and may reduce side effects, potentially through anti-inflammatory mechanisms [68,84]. In contrast, a retrospective study in patients with solid malignancies receiving Pembrolizumab reported worse overall survival among those using THC-containing CBS during treatment [110].

### 6.3. Translational Challenges

#### 6.3.1. Serum Influence on CBS Activity

In vitro studies have highlighted the complexity of CBD–chemotherapy interactions and raised important questions about the translation to clinical practice. Notably, it has been reported in vitro that serum levels appear to influence whether CBD potentiates or antagonizes the effects of chemotherapy. In triple-negative MDA-MB-231 cells, both serum concentration and protein binding significantly altered responses to combined CBS and chemotherapy treatment [52,136]. Studies reported that both CBD and THC have a high binding to plasma proteins, up to 98%, and the free circulating concentrations achievable in humans are likely to be substantially lower than the micromolar concentrations commonly used in vitro [136,137]. Additionaly, it raises a question of whether such concentrations can be reached at clinically safe doses. Research indicated that serum, particularly fetal bovine serum and albumin, can substantially reduce the cytotoxic potency of CBS. This effect has been observed in multiple cancer cell lines, including glioblastoma, melanoma, and colorectal cancer models [137]. In humans, serum and in vivo factors similarly influence CBD concentrations and pharmacological activity [138]. CBD is highly bound to plasma proteins, such as albumin, and only a small unbound fraction is pharmacologically active. This binding may become non-linear at high concentrations, further complicating predictions of in vivo efficacy. Thus, cytotoxic effects observed in vitro may not be achievable under physiological conditions in patients.

Taken together, although many clinical and real-world reports show that CBD can ameliorate treatment-related symptoms and its side effects [16,17,27,68,84], serum-dependent attenuation of CBD activity presents a potential barrier to clinical translation. These observations highlight the need for future studies designed to more accurately visualize potential interactions and improve predictions of safety, toxicity, and efficacy.

#### 6.3.2. Gap Between Preclinical and Clinical Evidence

A key limitation in CBD research is the translational gap between preclinical findings and clinical outcomes. Mechanistic studies are largely derived from 2D cell cultures and murine models, where exposure can be tightly controlled and endpoints are often short-term [27,29,30]. Moreover, apparent enhancement or synergistic effects observed in vitro may be influenced by experimental artifacts, including fixed drug concentrations, limited dose–response analyses, or assay-dependent readouts. While murine models capture systemic pharmacological and biological processes, which are absent in simplified in vitro systems, they still allow for standardized CBS administration, minimizing variability. However, significant interspecies differences in genetics, anatomy, physiology, and immune function limit the direct extrapolation of these findings to patients [139,140]. In clinical settings, patient exposure to CBS is substantially more variable, reflecting interindividual differences as well as reduced control over formulation, dosing, and treatment timing [141,142].

#### 6.3.3. Lack of BC-Specific Data for Certain CBS–Therapy Combinations

In BC, the literature on the combination of CBS with immunotherapy is extremely limited and largely inconclusive. Most available data come from preclinical models or clinical studies in heterogeneous cancer populations, and to date, no prospective clinical studies have specifically evaluated CBS use in BC patients receiving immune checkpoint inhibitors. Future research should prioritize prospective study designs to systematically assess dose, timing, and context-dependent effects of CBS in combination with immunotherapies such as Pembrolizumab. It would be beneficial to include large cohorts and determine which CBS is used and type of formulation. This would provide clinically relevant evaluation of CBS–therapy interactions.

In the CT26 (colon carcinoma) murine model, THC combined with the monoclonal antibody Pembrolizumab (monoclonal antibody used also in BC) did not compromise Pembrolizumab’s antitumor efficacy [101]. However, this model does not represent breast cancer biology. Retrospective cohort studies in patients with solid malignancies receiving immune checkpoint inhibitors have reported worse survival outcomes associated with concomitant THC-containing CBS use [98,110]. For instance, reduced response rates and shorter survival were observed in advanced cancer patients treated with immune checkpoint inhibitor Nivolumab who consumed predominantly THC-based products [98]. It is hypothesized that these deleterious effects of CBS are attributable to its recognized immunosuppressive properties [103,115,116]. Notably, studies linking CBS use to worse survival outcomes in cancer patients largely evaluated CBS as a single group, without differentiating between individual CBS, formulations or routes of administration [98,110]. But several studies have reported divergent findings depending on the type of CBS exposure, formulation, and experimental or clinical context [100,101,110]. For instance, in triple-negative MDA-MB-231 cells, CBD has been shown to enhance immune responses [100]. Therefore, given the distinct mechanisms and effects between individual CBS, these aggregated results should be interpreted with caution.

Importantly, no studies have examined the use of CBS alongside targeted therapies in BC, including tyrosine kinase inhibitors such as Tucatinib, Neratinib, and Lapatinib, or monoclonal antibodies such as Trastuzumab. These therapies are central to the management of HER2-positive BC, yet their potential interactions with CBS remain entirely unexplored. Research in this area is particularly important because CBS may modulate signalling pathways, affect treatment efficacy, or mitigate therapy-related toxicities.

#### 6.3.4. Formulation and Delivery

The administration of CBS presents a major challenge when attempting to translate in vitro preclinical findings to clinical settings. Substantial heterogeneity exists in both formulation strategies and delivery routes. Unlike preclinical models with controlled exposure, patients may consume CBS via diverse routes, including oral oils or extracts, inhalation (smoking or vaporization), or concentrated preparations, each associated with distinct pharmacokinetic profiles and exposure dynamics [143,144]. Even under a controlled environment, the variability in THC and metabolite levels are substantial between individuals [141].

Most in vitro studies employed free, solvent-dissolved CBS [30,33,71,73,83], while a smaller subset utilized nano- or carrier-based systems such as extracellular vesicles or polymeric microparticles [29,32]. These formulation differences are likely to influence CBS stability, cellular uptake, exposure duration, and intracellular distribution [145]. For instance, two in vitro studies reported dose-dependent differences in outcomes when non-psychoactive CBD and cannabinol were combined with chemotherapeutic or hormonal therapies [33,83]. High concentrations of CBD exhibited antagonistic effects when combined with doxorubicin in vitro [33], whereas low doses of cannabinol combined with exemestane were associated with attenuated ERα transcription [83].

#### 6.3.5. The Need for Clear and Specific Clinical Guidelines

Recent global data indicate that nearly 50 countries have fully or partially legalized CBS-based substances, reflecting a substantial expansion of legal access worldwide [146]. As the legalization of medical CBS continues to expand globally, the demand for robust, evidence-based clinical guidance is increasing [147,148,149]. Systematic reviews and narrative clinical analyses highlight considerable heterogeneity in CBS formulations, dosing strategies, and treatment durations across clinical studies, which currently precludes the development of clear and specific guidelines [147,148]. For instance, the inclusion of both oral and inhaled THC formulations in patients, as well as inclusion of patients with diverse solid malignancies and distinct disease biology, complicates mechanistic interpretation (e.g., of the observed negative interaction with immunotherapy). Notably, the regulatory and clinical guidance gaps are further compounded by inconsistent product labelling, with significant discrepancies between labelled and actual CBS content reported in edible medical CBS products [150,151]. The labelling and guidelines discrepancies could significantly affect patients’ safety, potentially leading to adverse cognitive effects and reduced psychomotor functions [152]. Additionally, studies indicate that such use may increase the risk of developing CBS use disorder, defined as a pattern of problematic CBS use associated with impaired control, tolerance, withdrawal, and continued use despite adverse consequences [153]. Labelling variability may hinder trial reproducibility by introducing uncertainty in CBS composition, dosing, and pharmacological effects [150,151].

### 6.4. Which CBS to Use?

A wide range of CBS have been identified. *Cannabis sativa* contains several major CBS (e.g., THC, CBD, CBG, cannabinol) and more than 120 minor CBS, which remain less researched and poorly understood [61]. While available in vitro and in silico studies highlight important differences among CBS subtypes, most experimental assessments focus on the major CBS and their individual effects [63,66]. Although all major CBS share immunomodulating properties, CBD has shown the greatest promise when combined with BC treatment in vitro [63]. These differences in effects may be explained by distinct mechanisms of action.

For instance, in receptor-based pharmacological assays and in vitro studies, CBD exhibited low affinity for CB1 and CB2 receptors and acted as a negative allosteric modulator of CB1, dampening CB1 signalling rather than driving it [154,155]. Inhibition of CB1 signalling has been shown to reduce mitochondrial dysfunction and inflammatory signalling in non-tumour in vivo models, whereas in vitro cancer studies demonstrate that CB1 signalling promotes tumour cell survival [40,156,157]. This suggests that dampening CB1 activity may negatively affect pro-survival pathways in cancer cells. Moreover, its antitumour profile is also supported by multiple-pathway actions: enhanced PPARA expression and cGAS-STING pathway in vitro, as well as inhibition of the NF-kB- pro-inflammatory pathway in both in vivo and in vitro settings [40,100,158]. In contrast to CBD, which promotes both autophagy and apoptosis, THC has been shown primarily to induce apoptotic cell death in BC cell lines [63]. While CBD act as a negative allosteric modulator of CB1, THC functions as a partial agonist of this receptor in vitro [159,160], potentially triggering downstream signalling that promotes cell survival [156,161].

In vitro studies of aromatase inhibitors show that combining CBD or cannabinol with irreversible aromatase inhibitors yields the most promising outcomes, whereas combinations with reversible aromatase inhibitors appear less effective [73,83]. This could be attributed to their nature of inhibition. Letrozole and Anastrozole are competitive inhibitors of aromatase, while Exemestane binds covalently to the aromatase enzyme, causing permanent inactivation [57,58]. A molecular docking study further suggested that Letrozole and Anastrozole may, under specific conditions, demonstrate non-competitive inhibition by modifying the inhibitory mechanism [162]. This could make them less stable when combined with CBS due to different conditions.

## 7. Conclusions

This review was conducted as a narrative synthesis of the available literature, without formal inclusion or exclusion criteria or structured risk-of-bias assessment. The aim was to provide a comprehensive overview of studies examining combinations of CBS with conventional BC therapies. Given the emerging and heterogeneous nature of the field, relevant studies identified through an extensive literature search were included without restriction by study design or experimental model.

Overall, in vitro and in vivo studies have yielded encouraging results regarding the combination of CBS with conventional cancer therapies. CBS have been suggested to act as adjuvant therapies and potential anti-cancer agents, exhibiting properties such as appetite stimulation, cancer cell proliferation inhibition, and induction of apoptosis [163,164]. They may also enhance the effects of conventional chemotherapies [45,165,166,167]. Notably, compounds such as THC and CBD have demonstrated the ability to modulate key cancer-related signalling pathways, contributing to tumour growth suppression across diverse preclinical experimental models [167]. Nevertheless, substantial limitations remain that hinder the translation of in vitro and in vivo findings into clinical practice. Although preclinical studies have reported promising results, clinical evidence remains scarce, and there is insufficient data on long-term safety, pharmacokinetics, and potential drug interactions. Consequently, no CBS–therapy combination is currently ready for clinical use in breast cancer treatment.

Further research should prioritize investigation of individual CBS, with the aim of elucidating their specific mechanisms of action when combined with BC therapy. If combinations with particular CBS prove effective, they may allow dose reduction of standard therapeutics, potentially decreasing treatment-related toxicity. Given the important role of targeted therapy and immunotherapy in BC, and the current scarcity of data on their combination with CBS, preclinical and clinical studies evaluating these combinations are urgently needed. Moreover, as variability among BC cells may result in differing responses to the same therapeutic combinations, future research should explore genetically informed personalized approaches to optimize treatment selection and outcomes.

## Data Availability

No new data were created or analyzed in this study.
